# A multi-objective optimization design approach of large mining planetary gear reducer

**DOI:** 10.1038/s41598-023-45745-5

**Published:** 2023-10-30

**Authors:** Wen Xin, Yanyan Zhang, Yang Fu, Wei Yang, Huanping Zheng

**Affiliations:** 1 CITIC Heavy Industry Machinery Co., Ltd, Luoyang, 471023 China; 2 National United Engineering Laboratory for Advanced Bearing Tribology, Henan University of Science and Technology, Luoyang, 471023 China; 3 College of Mechanical and Vehicle Engineering, Chongqing University, Chongqing, 400030 China; 4 State Key Laboratory of Intelligent Mining Heavy Equipment, Luoyang, 471023 China; 5 National Key Laboratory of High-End Equipment Mechanical Transmission, Chongqing, 400030 China

**Keywords:** Aerospace engineering, Mechanical engineering

## Abstract

A two-stage computational framework is proposed to optimize the radiated noise and weight of a large mining planetary gear reducer under the rated conditions, based on a combination of response surface methodology and multi-objective optimization. The well-established transient dynamic analysis model of a large mining planetary gear reducer, which is used to analyze the mechanical strength and acoustic characteristics of the gear reducer. A unified experimental design is developed to obtain the response surface of the gearbox radiated noise and the mass of the gearbox housing. After obtaining the multi-objective optimization function, the multi-objective optimization problem for a lightweight and low-noise gearbox is performed using non-dominated sorting from the Genetic Algorithm II (NSGA-II). The research results demonstrates the effectiveness of the proposed optimization method in reducing vibrating amplitude and weight of the gearbox. This is crucial for minimizing energy consumption and enhancing the overall performance of the system. Additionally, the optimized gearbox design not only saves energy but also contributes to the reduction of carbon emissions, making it environmentally friendly.

## Introduction

The power and level of modernization of large mining equipment are continuously improving. Because of the potential for pollution, laws, and regulations governing energy consumption, noise, and vibration are becoming more stringent^1–3^. The two-stage planetary reducer is an important component of the mining hoist and is crucial in tower transmission. It has a non-linear and complex mechanical system with multiple degrees of freedom, multiple gaps, and variable parameters. It exhibits a strong non-linear impact-dynamic contact phenomenon during operation, which is the primary cause of structural deformation, vibration, and radiated noise. This phenomenon affects the structural life and reliability of the equipment, as well as the physical and psychological health of the workers^4–6^. As a result, reducing radiated noise and weight are critical factors in the design of new large mining equipment. These optimization areas are of great interest to researchers and have received a lot of attention^7,8^.

In general, engineers have depended on their experimental experience to update their designs for improving a possible approach to overcome this difficulty is to apply a structural optimization method. Structural optimization has been applied to the design of automatic transmissions. Schönknecht et al.^9^ designed the Electric Powertrain of Battery Electric Vehicles with the methodology of multi-objective optimization. The optimization scope includes relevant electric powertrain components from the battery, inverter, and electric machine to the gearbox. Cao et al.^10^ proposed a novel parallel selection method of multi-objective optimization to obtain the optimal geometry, while the maximum finisher forming force and the maximum finisher die stress are both minimized. Wang et al.^11^ took cycloid speed reducers as the research subjective and provided an optimization methodology based on a genetic algorithm. The goal of the optimization is to simultaneously minimize the volume and maximize efficiency. The design variables including the short width coefficient, the diameter of the pin, the width of the cycloid gear, etc. are defined. The constraint conditions are established for multi-optimizations. Zhao et al.^12^ proposed a method to optimize gear parameters by reducing the transmission ratio. MATLAB genetic algorithm was adopted to optimize the design. The minimum volume of the rotary reducer was taken as the optimization design objective, and the design variables and constraints were determined. According to the calculated minimum strain energy, Slavov et al.^13^ optimized the overall weight of the reducer by refining the specific area of the shell element of the cast gearbox through the topology optimization algorithm.

Tamboli et al.^14^ researched the optimal design of a heavy-duty helical gear pair using particle swarm optimization. They presented the formulation of the constrained non-linear multi-variable optimization problem with derived objective functions and constraints. Choil et al.^15^ proposed structural optimization designs for lightweight and low-radiated noise automobile gearboxes based on modal reduction technology. In their study, the radiated-noise prediction for the gearbox was realized by calculating the velocity response of the finite element surface. The single-objective optimization design was essentially built and the thickness of the box was the design variable. The minimum mass of the vehicle transmission was the optimized objective function and the sound power was the constraint condition. Ide et al.^16^ proposed a design approach using the structural optimization method to minimize the radiated noise and reduce the mass of the outer casing of the automatic transmission. Three different structural optimization methods, topometry, topography, and freeform optimization, were applied. Their studies essentially resolved the single objective optimization problem.

Samad et al.^17^ carried out a multi-objective numerical optimization to optimize the total compression ratio and adiabatic efficiency of the blades of an axial compressor. Kimlt et al.^18^ built an optimum design method for an axial fan blade. They constructed the objective function based on an established response surface model and solved it using the NSGA-II algorithm. Wang et al.^19^ established a hybrid optimized design algorithm and optimized the rotor blade of a compressor. Miler et al.^20^ adopted genetic algorithm for multi-objective optimization of gear pair parameters, aiming at reducing transmission volume and power loss. Narayanan et al.^21^ introduced a new Many-objective Sine–Cosine Algorithm (MOSCA), which employs a reference point mechanism and information feedback principle to achieve efficient, effective, productive, and robust performance. Booth conducted a multi-objective shape optimization study of a multicellular aluminum extrusion subjected to dynamic axial and oblique (20° angle) impact loading conditions^22^. Li et al. proposed a two-step computational framework based on a combination of response surface methodology and multi-objective optimization to model the outlet air state of a desiccant wheel and subsequently optimize its operation^23^.

The demand for quality products has increased and environmental regulations are becoming more rigorous. To meet the demands of the market and users, researches must to carry out significant work on how to reduce the weight and radiated noise of the gearbox. However, there is little about these types of issues in the literature.

In the field of engineering, the parameters of the designed mechanical transmission system were not easily modified in the optimization research. Therefore, the proposed optimization plan which mainly starts from the transmission path of the dynamic load, which has two kinds load path. One is from the bearing to the gearbox and the other is from the planet gear engagement with the gear ring to the gearbox. In this study, we proposed a system-level transient dynamics analysis model using a hoist two-stage planetary gear reducer as the research object. The stress distribution law of each component under the rated conditions was obtained to determine components for weight reduction. The vibration velocity law of distribution was obtained using the transient dynamic calculation results as the load boundary conditions for the frequency response analysis of the gear box. The radiated noise of the gearbox and the contribution quantity of the acoustic panel were also obtained. A new optimization scheme was put forward for the vibration noise reduction and decreased weight of the large gearbox’s multi-objective optimization design.

## The mechanical strength analysis and acoustic prediction of the large mining planetary gear reducer

### The transient strength analysis of the two-stage hoist’s planetary gear reducer under the rated conditions

To establish the three-dimensional non-linear contacting dynamic model of the planetary gear reducer, the appropriate simplified treatments of the holes and bolts were performed under the condition that the calculation accuracy was not affected. The explicit three-dimensional solid hexahedral element and shell element were selected to mesh with the solid model of the two-stage hoist’s planetary gear reducer and took into consideration the finite element model scale and requirements for the calculation’s accuracy. The rotating speed and resisting moment were applied on the shell element.

**Figure 1:**
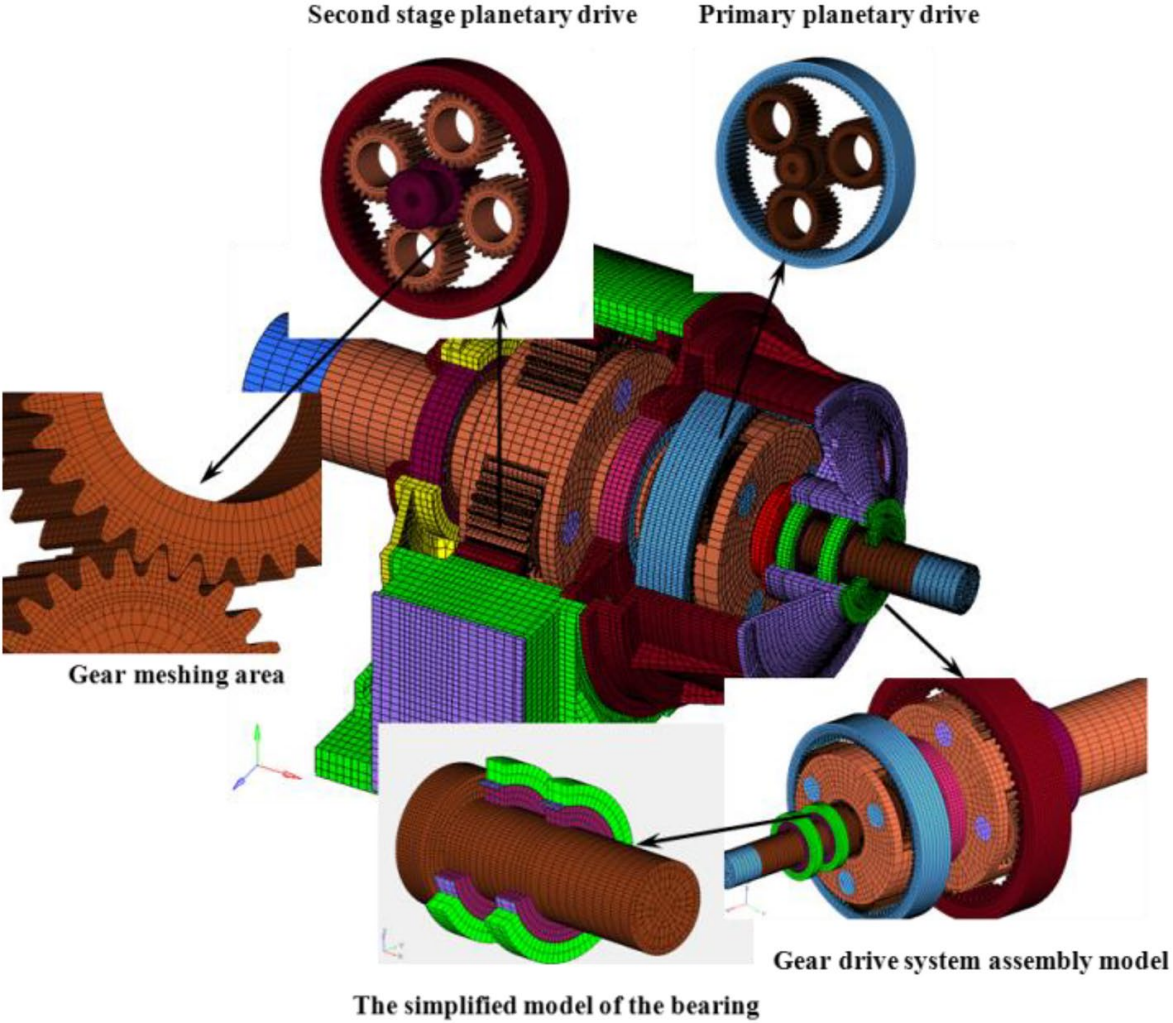
A system-level non-linear dynamic model of the hoist equipped with a two-stage planetary gear transmission system.

A transient dynamics model is obtained to restore the nonlinear forces and moments acting on the through the bearings of the transmission system to a greater extent. Figure 1 shows the system-level non-linear dynamic model of the hoist equipped with a two-stage planetary gear transmission system. The detailed theoretical analysis can be found in the literature^24^.

A rigid surface is established on the input and output shaft ends to force the loads. As shown in Table 1, the rotation input shaft end is a constant speed of 740r/min, which is applied to the auxiliary rigid body surface of the input shaft end. The power of the output shaft end is a constant resistance moment 380000N.m, which is applied to the auxiliary rigid body of the output shaft end. When the speed and resistance moment are applied and increased linearly from zero from 0 s, stabilize to a constant value at 0.01 s, and then remain constant. The box base is restrained to simulate the component constraints under the actual working conditions of the reducer. In addition, based on the geometric characteristics and different functions of the bearings in the system, some degrees of freedom of the bearings are constrained.

**Table 1:** Parameters of the two-stage hoist’s planetary gear reducer.

Power	Output torque	Input speed	Transmission ratio
1400 kW	380000 N m	740 rpm	21

**Figure 2:**
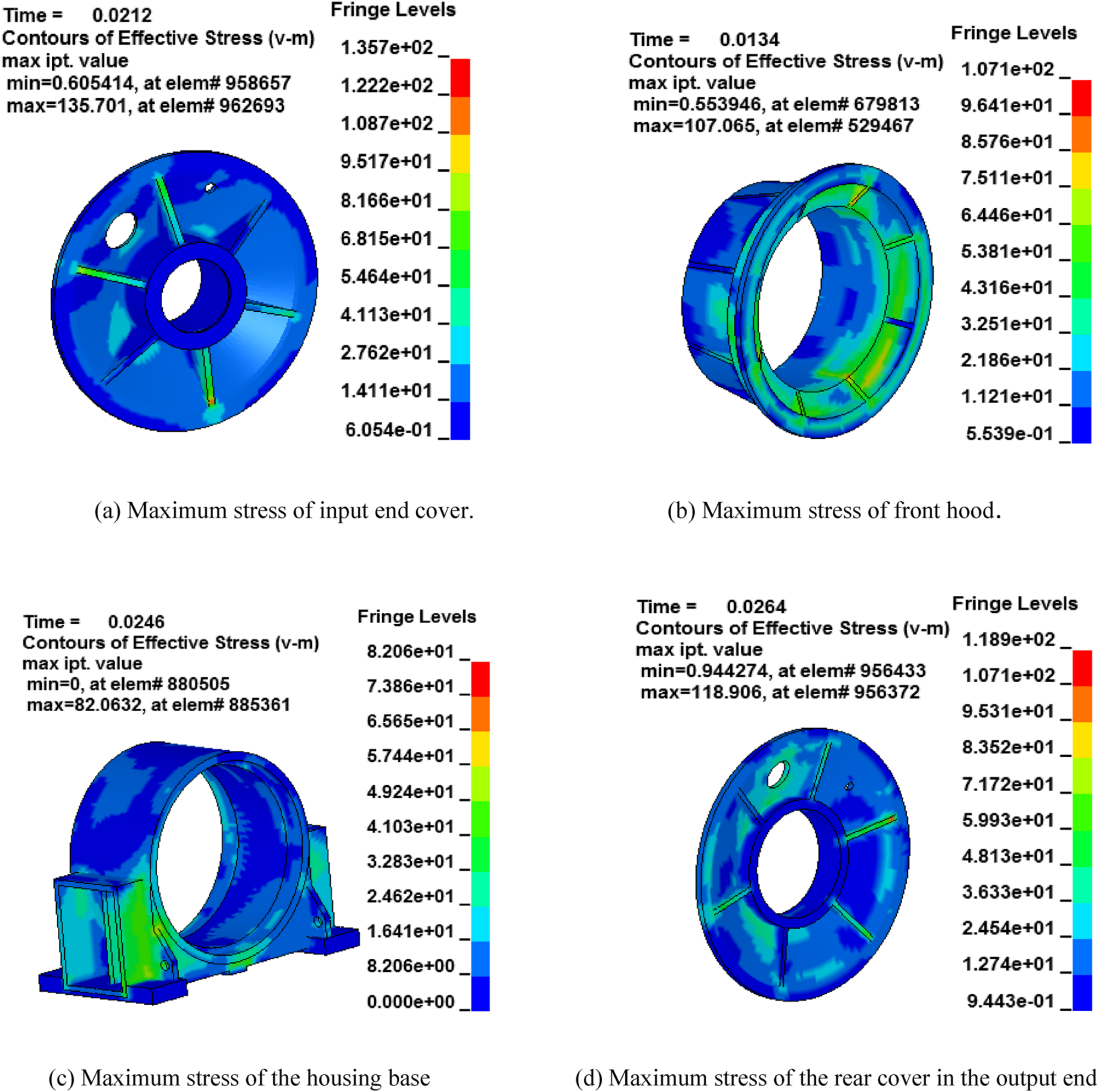
The transient dynamic Stress nephograms of some components in the reducer box.

Figure 2 depicts the transient dynamic stress nephograms of some components in the reducer box obtained simulation results on the LS-DYNA software platform. In general, the stress is concentrated in the nearby regions supporting the housing bearing's block hole, the housing body stiffener, the housing near the ring gear junction, and other areas. The stiffener rib of the end housing and bearing seat is under the most stress. The maximum stresses of the following parts were found to be lower than their yield limits, housing base, small rear end cover, rear end housing, side box, and front-end cover. The housing base, front end cover, and small front-end cover are massive parts.

The maximum stress, materials’ properties, and yield stress of the two-stage planetary reducer under the rated working conditions are listed in Table 2. Under rated operating conditions, components with stress values lower than the yield limit of the material and a significant proportion of weight will be selected as the main object of weight reduction.

**Table 2:** Mass and the mechanical properties of the materials of the two-stage planetary reducer.

	Material	Yield limit $\sigma_{s} \;({\text{Mpa}})$	Maximum stress $\sigma_{\max } \;({\text{Mpa}})$	Mass $M\;({\text{Kg}})$
Rear end cover	Q235A	235	118.9	643.2
Small rear end cover	Q235A	235	62	90.45
Housing base	HT200	310	82.1	4931
Left and right end covers	Q235A	235	40	185.5
Front hood	HT200	310	107.1	1525.0
Front end cover	Q235A	235	135.7	498.5
Small front end cover	Q235A	235	31	43.73
Input shaft	42CrMo	930	917	183.4

### Vibration test and analysis of the two-stage hoist’s planetary gearbox

#### Vibration test of the gear box

**Figure 3:**
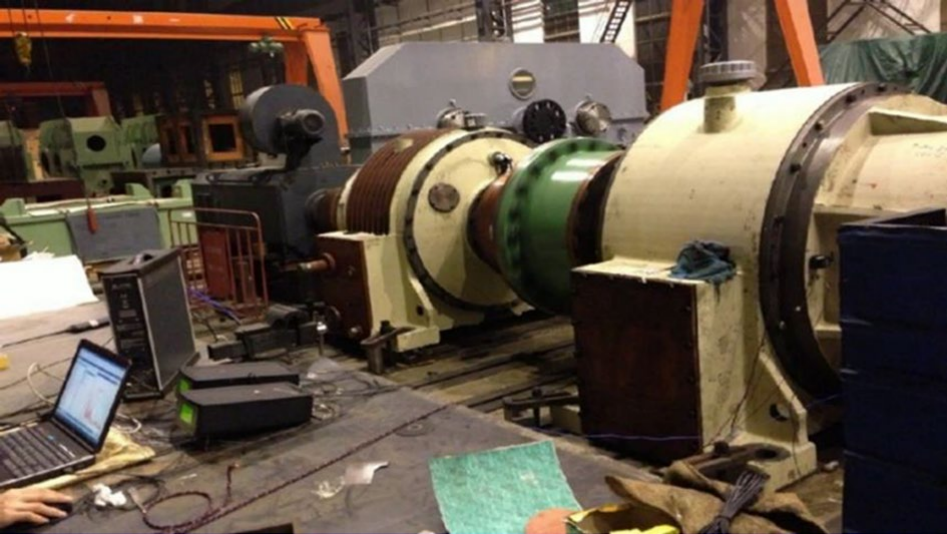
Test rig of the two-stage planetary reducer.

To verify the correctness of the transient dynamic model, vibration testing was carried out. Six measuring points were selected based on theoretical results. Measuring point 1 is fixed on the left end of the front cover near the primary gear ring, measuring point 2 is laid on the top end of the end cover near the bearing seat at the input end, measuring point 3 is arranged on the left end of the end cover near the bearing seat at the input end, measuring point 4 is installed on the left stiffener of the frame, measuring point 5 is mounted on the top end of the frame near the secondary gear ring, and measuring point 6 is arranged on the top end of the end cover near the bearing seat at the output end. The vibration and noise tests of the hoist reducer were conducted on the CITIC Heavy Industry gearbox vibration test bench, as shown in Fig. 3. Due to the rated power of the reducer being 1400kw and its large volume. In the experiment, a servo motor is used to drive the reducer, and an identical reducer is connected to the output end to load the tested reducer. The sound field is 200 mm from the vertical distance of each component.

#### Experiment results and discussion

**Figure 4:**
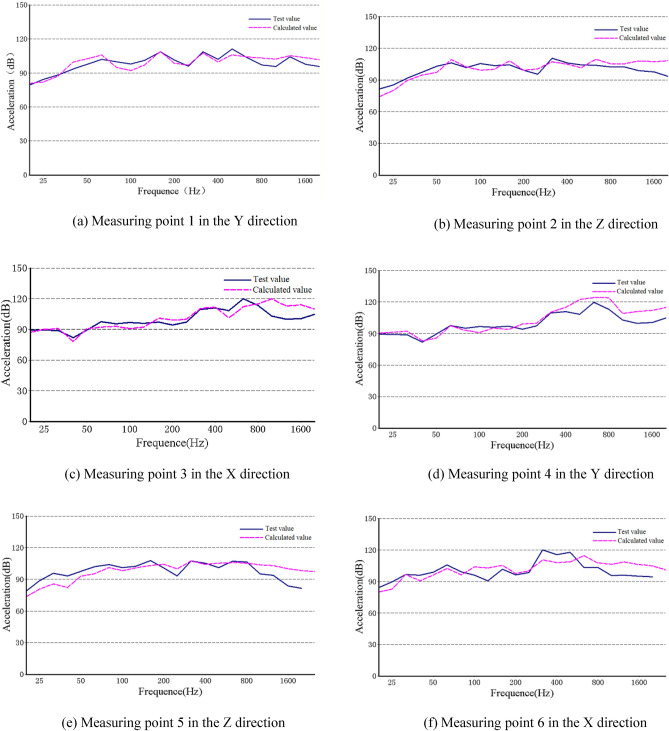
Comparison of structural noise between the experiment results and the simulation calculation results of the measuring points.

Figure 4 depicts comparison curves between the experiment results of acceleration level 1/3rd octave structural noise at 500 rpm and 20% rated load, and the simulation calculation results. The calculated results are in good agreement with the test results in the low frequency band, and the calculated value is larger than the test value in the high frequency band. It is mainly due to the simplification of various damping components of the actual reducer in the calculation model, and the experimental error caused by the condition limitation in the experimental test. But the overall trend is consistent. The experiment results are proved the correctness of the three-dimensional transient dynamic model.

### The acoustic prediction analysis of the two-stage hoist’s planetary gear box under the rated working conditions

#### Panel contribution analysis of the gear box body

**Figure 5:**
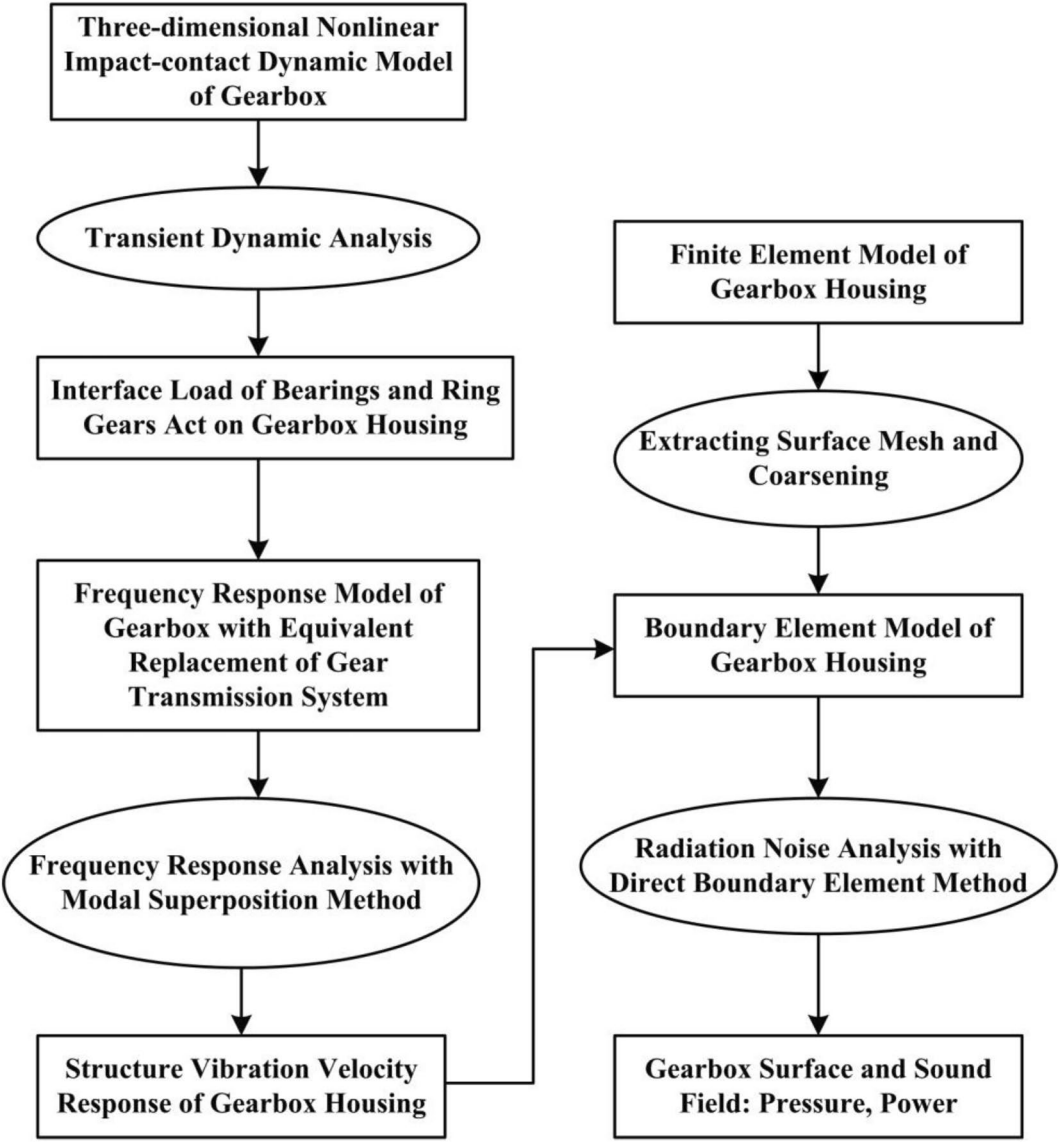
The schematic of the acoustic prediction for the planetary gearbox.

In operation, the radiated noise of the two-stage planetary gear reducer is generated from the vibration velocity. The radiated noise of the gearbox housing was researched based on the acoustic boundary element model, and the field-point mesh of the box where the calculation result of the velocity of frequency response was used as the input parameter of boundary condition. The schematic of the analysis is shown in Fig. 5. The boundary condition of the sound pressure $\Omega_{p}$ indicates that the sound pressure solution, $p(\vec{r})$ of the Helmholtz equation, needs to meet the following requirement:

1 $$ p(\vec{r}) = \overline{p}(\vec{r}),\vec{r} \in \Omega_{p} $$

where $\overline{p}(\vec{r})$ is the sound pressure on the velocity boundary.

For the velocity boundary condition, $\Omega_{v}$ , the sound pressure solution $p(\vec{r})$ of the Helmholtz equation needs to satisfy the following equation

2 $$ v_{n} (\vec{r}) = \frac{j}{{\rho_{0} \omega }}\frac{{\partial p(\vec{r})}}{\partial n} = \overline{v}_{n} (\vec{r}),\vec{r} \in \Omega_{v} $$

where $n$ is the normal direction of the boundary,$\overline{v}_{n} (\vec{r})$ is the normal velocity on the velocity boundary,$\omega$ is the angular frequency, and $\rho_{0}$ is the fluid density.

For the boundary conditions of the acoustic impedance,${\Omega }_{z}$, and the sound pressure solution $p(\vec{r})$ of the Helmholtz equation needs to meet the following conditions

3 $$ p(\vec{r}) = \overline{Z}(\vec{r}) \cdot v_{n} (\vec{r}),\vec{r} \in \Omega_{z} $$

where $\overline{Z}(\vec{r})$ is the acoustic impedance on the boundary of the acoustic impedance.

The external sound field, such as the infinite boundary condition, $\Omega_{\infty }$ must be taken into account during the sound pressure solution $p(\vec{r})$ of the Helmholtz equation

4 $$ \mathop {\lim }\limits_{{\vec{r} \to \infty }} \left| {\vec{r}} \right| \cdot \left( {\frac{{\partial p(\vec{r})}}{{\partial \left| {\vec{r}} \right|}} + jkp(\vec{r})} \right) = 0 $$

In general, the sound pressures and vibration velocities of some nodes are unknown in the direct boundary element. The unknown node information needs to be calculated through the known node information. The following equation indicates the unknown nodes

5 $$ A_{b} \left\{ {p_{i} } \right\} = j\rho_{0} \omega B_{b} \left\{ {v_{ni} } \right\},\;{\text{for}}\;b = 1,2, \ldots ,n_{a} $$

where,$A_{b}$ and $B_{b}$ are the coefficient matrixes for a and b different nodes, respectively.

By applying the Green function,$G\left( {\vec{r}} \right) = \frac{{e^{{ - jk\vec{r}}} }}{{4\pi \vec{r}}}$, The elements of the previously mentioned coefficient matrices (a and b) can be written as

6 $$ A_{bi} = \delta_{bi} \left( {1 + \frac{1}{4\pi }\int {\frac{\partial }{\partial n}\left( {\frac{1}{{\left| {\vec{r}_{b} - \vec{r}_{a} } \right|}}} \right)} \cdot d\Omega (\vec{r}_{a} )} \right) - \int\limits_{{\Omega_{a} }} {N_{i} (\vec{r}_{a} )\frac{{\partial G(\vec{r}_{a} ,\vec{r}_{b} )}}{\partial n}} \cdot d\Omega (\vec{r}_{a} ) $$

7 $$ B_{bi} = \int\limits_{{\Omega_{a} }} {N_{i} (\vec{r}_{a} )G(\vec{r}_{a} ,\vec{r}_{b} )} \cdot d\Omega (\vec{r}_{a} ) $$

where $$\delta_{bi} = \left\{ {\begin{array}{*{20}c} {0,} & {b \ne i} \\ {1,} & {b = i} \\ \end{array} } \right.$$,

By Eqs. (1)–(7), the relationship between the sound pressure and vibration velocity for all unknown nodes on the boundary element mesh $\Omega_{a}$ can be got from the following equation

8 $$ A\left\{ {p_{i} } \right\} = j\rho_{0} \omega B\left\{ {v_{ni} } \right\} $$

where $A$ and $B$ are comprised of $A_{a}$ and $B_{b}$.

In the sound field,$V$,the sound pressure,$p(\vec{r})$ at any point located outside of boundary element can be shown by the integral between the sound pressure $\left\{ {p_{i} } \right\}$ and normal vibration velocity $\left\{ {v_{ni} } \right\}$ on the boundary element

9 $$ p(\vec{r}) = \left\{ {C_{i} } \right\}^{T} \left\{ {p_{i} } \right\} + \left\{ {D_{i} } \right\}^{T} \left\{ {v_{ni} } \right\},\;\vec{r} \in V\;{\text{and}}\;\vec{r} \notin \Omega_{a} $$

where $$C_{i} = \int\limits_{{\Omega_{a} }} {N_{i} (\vec{r}_{a} )\frac{{\partial G(\vec{r},\vec{r}_{a} )}}{\partial n}} \cdot d\Omega (\vec{r}_{a} ),\;D_{i} = j\rho_{0} \omega \int\limits_{{\Omega_{a} }} {N_{i} (\vec{r}_{a} )G(\vec{r},\vec{r}_{a} )} \cdot d\Omega (\vec{r}_{a} ),$$ $$i = 1,2, \ldots ,n_{a}$$, $$\vec{r} \in V$$ and $$\vec{r} \notin \Omega_{a} .$$

The visual and acoustic equation is linear under the small disturbance condition. A linear relationship was established between the sound pressures obtained from the location points of the structural surface vibration and the sound field.

After processing Eqs. (8) and (9) with the Fourier transform, the following equations were obtained:

10 $$ A(\omega )p_{i} = B(\omega )v_{ni} (\omega ) $$

11 $$ p = C^{T} p_{i} + D^{T} v_{ni} (\omega ) $$

The following relationship can be obtained from Eqs. (10) and (11)

12 $$ p = \left\{ {C^{T} A^{ - 1} (\omega )B(\omega ) + D^{T} } \right\} \cdot v_{ni} (\omega ) $$

Let $\left\{ {ATV(\omega )} \right\}^{T} = C^{T} A^{ - 1} (\omega )B(\omega ) + D^{T}$, where $ATV\;(\omega )$ is the acoustic transfer vector.

Equation (12) shows that the sound pressure of the sound field is obtained by multiplying the acoustic transfer vector and normal velocity of the vibrations. If the vibration structure is divided into several regions, the contribution of the sound pressure made by some of the vibration areas on some of the field points in the sound field is shown by the following equation

13 $$ p_{e} = \sum\limits_{e = 1}^{n} {\left\{ {ATV^{e} (\omega )} \right\}^{T} \cdot v_{ni}^{e} (\omega )} $$

where $p_{e}$ is the contribution of the sound pressure, and $n$ is the amount of the finite elements in this region.

The general acoustic power radiated out by the sound source is obtained from Eq. (14).

14 $$ W = \sum\limits_{i = 1}^{n} {W_{i} } ,\;i = 1,2, \ldots ,n $$

where, $W_{i}$ is the acoustic power produced at the $i$ th panel.

The panel contribution coefficients $T_{i}$ of the acoustic power are defined to describe the proportion that the acoustic power produced by the panel occupies in the total acoustic power of the sound field. Then, the contribution coefficient of the sound power of the panel with a frequency of $\omega$ is given as follows

15 $$ T_{i} (\omega ) = \frac{{W_{i} (\omega )}}{W(\omega )} $$

**Figure 6:**
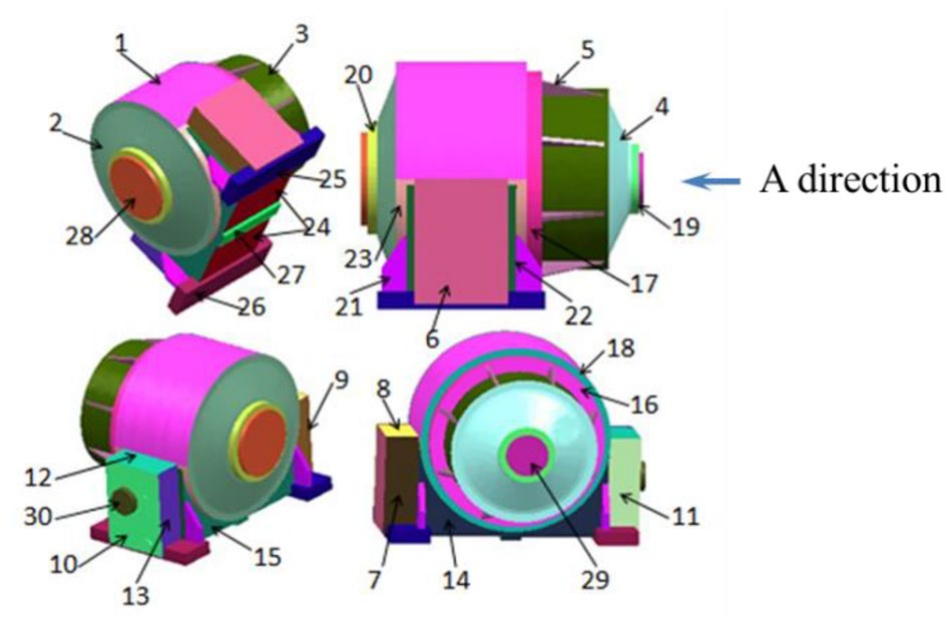
Panels of the boundary element area. Panel 1: The upper half circle arch of the housing; Panel 2: the rear end cover; Panel 3: the front hood; Panel 4: the front end cover; Panel 5: the rib-plate of the front hood; Panel 6: The left end face on the left side of the housing base; Panel 7: the front end face on the left side of the housing base; Panel 8: the top end face on the left side of the housing base; Panel 9: the rear end face on the left side of the housing base; Panel 10: the right end face on the right side of the housing base; Panel 11: the front end face on the right side of the housing base; Panel 12: the top end face on the right side of the housing base; Panel 13: the rear end face on the right side of the housing base; Panel 14: the front end face of the lower half of the housing base; Panel 15: the rear end face of the lower half of the housing base; Panel 16: the bottom face of the front hood; Panel 17: the bottom ring face of the front hood; Panel 18: the front circular face of the housing base; Panel 19: the annular convex face of the front end cover; Panel 20: the annular convex face of the rear end cover; Panel 21: the rib of the housing base; Panel 22: the strip surface of the lower half of the housing base; Panel 23: the circular surface of the lower half of the housing base; Panel 24: the bottom face of the gear box; Panel 25: the left bracket of the gear box; Panel 26: the right bracket of the gear box; Panel 27: the rib of the box bottom face; Panel 28: the face of the output end; Panel 29: the face of the input end; Panel 30: the right convex surface of the base structure.

The direct boundary element method (BEM) is used in LMS-Virtual Lab Acoustics software platform to simulate the radiation noise of the two-stage planetary gear reducer box. After analyzing the radiation noise level in the planetary gearbox, an acoustic panel contribution analysis was conducted. Based on its geometric characteristics, the gearbox was divided into 30 panels to determine the degree of influence of each component on the overall radiation noise. The distribution of panels is shown in Fig. 6. Considering the analysis results of transient dynamic stress, identify components with abundant strength and high radiation noise level as the optimization objects.

**Figure 7:**
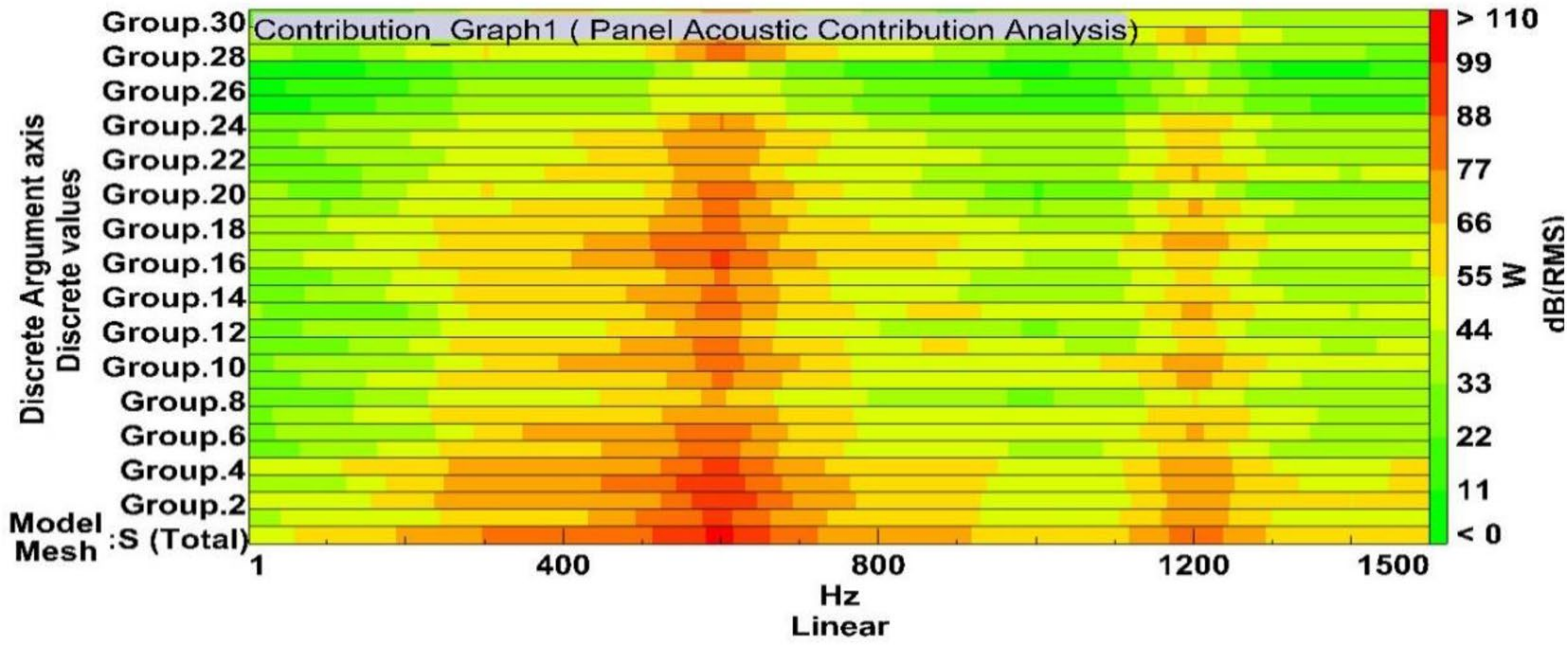
The sound pressure contribution of 30 panels to the sound power level.

Figure 7 depict that the contribution of the reducer box’s acoustic power in frequency domain mainly focused on the range from 400 Hz to700 Hz and around 1200 Hz. Its peak lies at a maximum at frequency of 600 Hz. According to the research results, the major vibration frequency is mostly excited by the meshing frequency of the planetary transmission frequency multiplication and/or doubling frequency. The mechanical structural strength of the studied object was more redundant, but the radiated noise to the environment exceeds the noise level that the operator can withstand. There is a great need for a lightweight structure in multi-objective optimal design.

The continuous experimental evaluation of the sample point set in the sub-domain of the design variables was performed using the experimental design. The goal of this study is to build a bridge between the design variables and optimization objectives. Few test points were needed to obtain the same precision with a higher calculation efficiency. This approach resolves the problem which needs a large amount of calculations, using the boundary element method to obtain the acoustic properties of the planetary gear box in a large hoist under the rated working conditions. Accordingly, the uniform experimental method was selected to conduct the experimental design.

## The uniform experiment

### Theory basis

The expressions of the optimization objective, constraint function, and design variable function can be obtained using the experimental design method. One of the core components of this approach is to select the test points of the random input variables. The uniform design is a kind of experimental design method where uniformly distributed test points are considered.

#### Sampling calculation of the multi-dimensional integral

The uniform design is based on the pseudo-Monte Carlo method of numerical integration, and its regression model can be expressed as

16 $$ y = g\left( {x_{1} ,x_{2} , \ldots ,x_{s} } \right) + \varepsilon $$

where the test area of $x_{s}$ is $C^{n} = \left[ {0,1} \right]^{s}$, where $g$ represents the known function class.

The average response test area,$C^{n}$ ,can be estimated from the following equation

17 $$ Avg\left( g \right) = m\left( g \right) + Avg\left( \varepsilon \right) \approx m\left( g \right) = \mathop \smallint \limits_{0}^{1} \cdots \mathop \smallint \limits_{0}^{1} g\left( {x_{1} , \ldots ,x_{s} } \right)dx_{1} \cdots dx_{s} = \mathop \smallint \limits_{{C^{S} }}^{{}} g\left( x \right)dx $$

The average value of the samples was adopted to replace the analytical expression of the function. For example, if $n$ times of the test were performed in the test area, and the test points can be shown as $p_{n} = \left\{ {x_{k} = \left( {x_{k1} ,x_{k2} , \cdots ,x_{ks} } \right),k = 1, \cdots ,n} \right\}$ , then the mean value of the function,$g$ at $n$ points is expressed as

18 $$ \overline{g} = \frac{1}{n}\mathop \sum \limits_{k = 1}^{n} g\left( {x_{k1} , \ldots ,x_{ks} } \right) $$

$m\left( g \right)$ can be estimated using Eq. (18).

#### The Koskma-Hlawka inequality

The Koskma-Hlawka inequality is expressed in Eq. (19), which gives the error range of the function approximation

19 $$ \left| {Avg\left( g \right) - \overline{g}} \right| \le V\left( g \right)D\left( {p_{n} } \right) $$

where $C^{S}$ is the total variation of function $g$ on $C^{S}$ ,$D\left( {p_{n} } \right)$ is the star discrepancy of the point set on $C^{S}$ ,$Avg\left( g \right)$ is the approximate value.

### Measure of uniformity

The estimation of the uniformity was determined using the deviation. By setting $p = \left\{ {x_{k} |k = 1, \cdots ,n} \right\}$ , i.e.,$n$ test points in $C^{S}$, and the function $g \in C^{S}$ by setting $N\left( {g,p} \right)$ as the number of test points dropped in the range of $g \in C^{S}$ , then the equation for the uniformity is

20 $$ D\left( {n,p} \right) = {}_{{g \in C^{S} }}^{\sup } \left| {\frac{{N\left( {g,p} \right)}}{n} - v\left( {\left[ {0,g} \right]} \right)} \right| $$

where $D\left( {n,p} \right)$ is the deviation of point $\rho$ in $C^{S}$ . $v\left( {\left[ {0,g} \right]} \right)$ presents the volume of a rectangle formed by $s$ points, and sup means is the upper limit. If a uniformly distributed $p$ is the input, the differences between $\frac{{N\left( {g,p} \right)}}{n}$, $v\left( {\left[ {0,g} \right]} \right)$, and the deviation will be smaller.

### Construction of the uniform design table

The good grid method was applied to determine the Uniform Table,$U_{n} \left( {q^{t} } \right)$ , the mesh method was a square matrix with $q$ rows and $t$ columns. Each row was a subset of $\left\{ {1,2, \cdots ,q} \right\}$ while each column is a substitution of $\left\{ {1,2, \cdots ,q} \right\}$ . The Uniform Table was determined using the following steps and methods.

Step 1 provided the test number,$n$ and found the integer,$h$,which is less than the integer $n$ ,to ensure that the maximum common divisor between $n$ and $h$ was 1. All the positive integers which met this condition comprised the vector $H = \left( {h_{1} ,h_{2} , \cdots ,h_{m} } \right)$ . where $m$ is the function of $n$ . This function can be expressed by the Euler function, $m = E\left( n \right)$ .

Step 2 represented a row of the Uniform Table from the obtained vector $H$, while $j$ was the column of the Uniform Design Table.

21 $$ u_{ij} = ih_{j} \left[ {modn} \right] $$

where $u_{ij}$ is the element of the $i$ th row and $j$ th column in the Uniform Design Table. $\left[ {modn} \right]$ is the operation of congruence. If $ih_{j}$ is over $j$ , it can be used to subtract the suitable multiple of $n$ so that the difference is in $\left[ {1,n} \right]$ .

The radiated noise and box mass of the gear box were used as variables. The structural positions, Panels 1–4 were also considered as experimental factors since the maximum noise is found in those panels. Panel 1 and Panel 3 were along the radial thickness variation, and Panel 2 and Panel 4 were along the axial thickness variation of the input and output. The experimental factors and spaces of the uniform design are listed in Table 3. The negative and positive values indicate the increment and decrease of the structural thickness, respectively. According to the principle of uniform design, many experiments for the 4 factors need to be performed. Three times the number of the experimental factors is considered an appropriate amount for the test data, since this amount is beneficial to the modeling and optimization. The uniform design table and application table of U31*(3110) were selected for the experimental design. The uniformity deviation of the 4-factor test was D = 0.11. During the experiments, the obtained sample points were substituted into the transient dynamic analysis model (As shown in Fig. 1), which provided that the sample points in the experimental design space were formed by each factor. The responses of the radiated noise and box mass for the sample points in each group were found using the analysis described in “Experiment results and discussion”. The obtained sample points and experimental results of the uniform experiment are listed in Table 5.

**Table 3:** The uniform experimental design of some index.

The level number of each factor is equal						
Name of Index 1: The unit of the maximum sound radiated pressure in the field points, dB (A)						
Name of Index 2: The unit of the box mass: kg						
Name of Factor 1: The thickness variable unit of the upper half of the structure of the housing, mm						
Name of Factor 2: The thickness variable unit of the rear cover housing in the output end, mm						
Name of Factor 3: The thickness variable unit of the cylindrical wall structure in the front seat of base, mm						
Name of Factor 4: The thickness variable unit of the front cover housing in the input end, mm						
No	Factor 1	Factor 2	Factor 3	Factor 4	Index 1	Index 2
1	0.600	− 0.900	8.000	5.200	101.18	7463
2	1.500	3.000	3.600	4.200	103.55	7460
3	2.400	− 2.700	12.00	3.200	101.27	7425
4	3.300	1.200	7.600	2.200	102.70	7422
5	4.200	− 4.500	3.200	1.200	101.66	7538
6	5.100	− 0.600	11.60	0.200	101.93	7384
7	6.000	3.300	7.200	5.600	103.55	7332
8	6.900	− 2.400	2.800	4.600	101.69	7447
9	7.800	1.500	11.20	3.600	102.68	7294
10	8.700	− 4.200	6.800	2.600	101.36	7408
11	0.000	− 0.300	2.400	1.600	102.47	7560
12	0.900	3.600	10.80	0.600	103.55	7405
13	1.800	− 2.100	6.400	6.000	101.17	7471
14	2.700	1.800	2.000	5.000	103.08	7469
15	3.600	− 3.900	10.40	4.000	101.23	7433
16	4.500	0.000	6.000	3.000	102.33	7430
17	5.400	3.900	1.600	2.000	104.64	7429
18	6.300	− 1.800	10.00	1.000	101.68	7392
19	7.200	2.100	5.600	0.000	103.85	7390
20	8.100	− 3.600	1.200	5.400	101.40	7455
21	9.000	0.300	9.600	4.400	102.29	7302
22	0.300	4.200	5.200	3.400	104.05	7452
23	1.200	− 1.500	0.800	2.400	102.15	7568
24	2.100	2.400	9.200	1.400	103.11	7414
25	3.000	− 3.300	4.800	0.400	101.69	7529
26	3.900	0.600	0.400	5.800	102.63	7477
27	4.800	4.500	8.800	4.800	104.07	7323
28	5.700	− 1.200	4.400	3.800	102.00	7439
29	6.600	2.700	0.000	2.800	104.26	7437
30	7.500	− 3.000	8.400	1.800	101.46	7400
31	8.400	0.900	4.000	0.800	103.51	7398
32	2.1335	4.3244	4.4217	5.8297	103.20	7356
33	0.1696	0.3987	7.2706	3.0939	101.73	7422
34	8.7714	1.7156	9.7028	1.3011	102.37	7318
35	3.7559	− 2.6235	6.0351	0.2494	103.44	7461
36	6.0618	− 4.4368	0.4793	3.6486	101.59	7425

## Approximate model of the response surface

Since the uniform experimental design only has the characteristics of the uniform dispersion, the experimental results in Table 3 can’t be analyzed using the variance analysis method. In order to solve this problem, the quadratic polynomial progressive regression analysis was adopted to separately build the approximate model of the response surface for the maximum radiated noise of the field points and box mass. The model is expressed below

22 $$ f\left( x \right) = a_{0} + \mathop \sum \limits_{i = 1}^{n} a_{i} x_{i} + \mathop \sum \limits_{i = 1}^{n} a_{ii} x_{i}^{2} + \mathop \sum \limits_{j < i}^{n} a_{ij} x_{j} x_{i} $$

where $a_{0}$ is the unknown constant, while $a_{j}$, $a_{ii}$ , $a_{ij}$, and $a_{i}$ are the unknown coefficients. $n$ is the number of design variables.

### Sound radiated pressure of approximate response surface

The quadratic polynomial approximate response surface of the radiated noise was obtained through the stepwise regression analysis under the following conditions. At the significance level α = 0.05, the critical value of the introduced variable,$F_{\alpha } = 4.543$, and the critical value of the removed variable, $F_{e} = 4.494$ were set. The expression can be expressed as

23 $$ Y_{dB} = {\text{a}}_{0} + {\text{a}}_{1} \cdot x_{2} + {\text{a}}_{2} \cdot x_{1} x_{1} + {\text{a}}_{3} \cdot x_{1} x_{2} + {\text{a}}_{4} \cdot x_{2} x_{2} + {\text{a}}_{5} \cdot x_{2} x_{3} + {\text{a}}_{6} \cdot x_{3} x_{3} {\text{ + a}}_{7} \cdot x_{3} x_{4} + {\text{a}}_{8} \cdot x_{4} x_{4} $$

where, the regression coefficients were $a_{0} = 102.59$, $a_{1} = 0.31230$, $a_{2} = 5.9523{\text{e}} - 3$, $a_{3} = 2.0355{\text{e}} - 2$, $a_{4} = {3}.{\text{5474e}} - {2}$, $a_{5} = - {6}.{624}0{\text{e}} - {3}$, $a_{6} = - {3}.{643}0{\text{e}} - {3}$, $a_{7} = - {9}.{6}0{\text{99e}} - {3}$, and $a_{8} = - 1.0380{\text{e}} - 2$.

**Figure 8:**
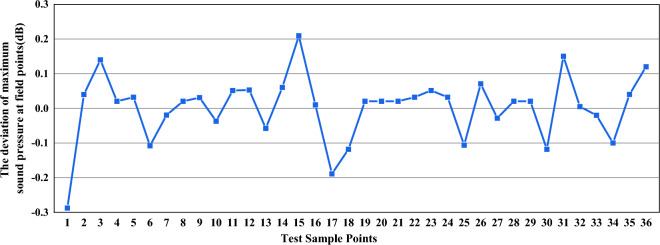
The deviation between the real value and approximate value of the maximum sound pressure at the field points.

From Fig. 8, it is evident that the real values obtained from the calculation are coincident with the approximate response values, and the deviation is small. Consequently, this confirms that the proposed approximate model of the response surface for the radiated noise and sound pressure can be used to replace the real response surface model of the sound pressure.

### The gear-box mass's approximate response surface

Similar to the method in “Approximate model of the response surface”, the quadratic polynomial approximate response surface of the box mass was obtained through the stepwise regression analysis that was expressed as

24 $$ M_{mass} = {\text{b}}_{0} + {\text{b}}_{1} \cdot x_{1} + {\text{b}}_{2} \cdot x_{2} + {\text{b}}_{3} \cdot x_{3} {\text{ + b}}_{4} \cdot x_{4} + {\text{b}}_{5} \cdot x_{1} x_{2} + {\text{b}}_{6} \cdot x_{3} x_{3} + {\text{b}}_{7} \cdot x_{3} x_{4} $$

where, the regression coefficient $b_{i}$ was $b_{0} = {7}.{5976}$, $b_{1} = - {1}.{\text{5929e}} - {2}$, $b_{2} = - {1}.{235}0{\text{e}} - {2}$, $b_{3} = - {1}.{\text{2123e}} - {2}$, $b_{4} = - {7}.{95}00{\text{e}} - {3}$, $b_{5} = {2}.{\text{2586e}} - {5}$, $b_{6} = {1}.{\text{7172e}} - {5}$, and $b_{7} = {2}.{5}0{3}0{\text{e}} - {5}{\text{.}}$

**Figure 9:**
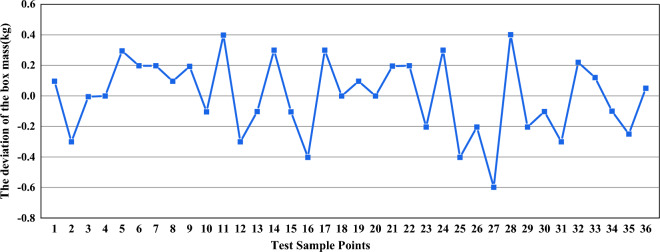
The deviation between the real mass of the gear box and the box mass calculated.

The error range between the mass obtained using the approximate model of the response surface and real mass is from 0 to 0.6 kg, as shown in Fig. 9. For a large gear box, its mass is nearly14,680 kg, and the occupied proportion of the error values is rather small. Thus, it can be said that the approximate response surface model of the box mass can accurately approach the real response surface of the housing mass.

## The multi-objective optimization method of the large planetary gear box

### The multi-objective optimization function of the large planetary gear box

**Figure 10:**
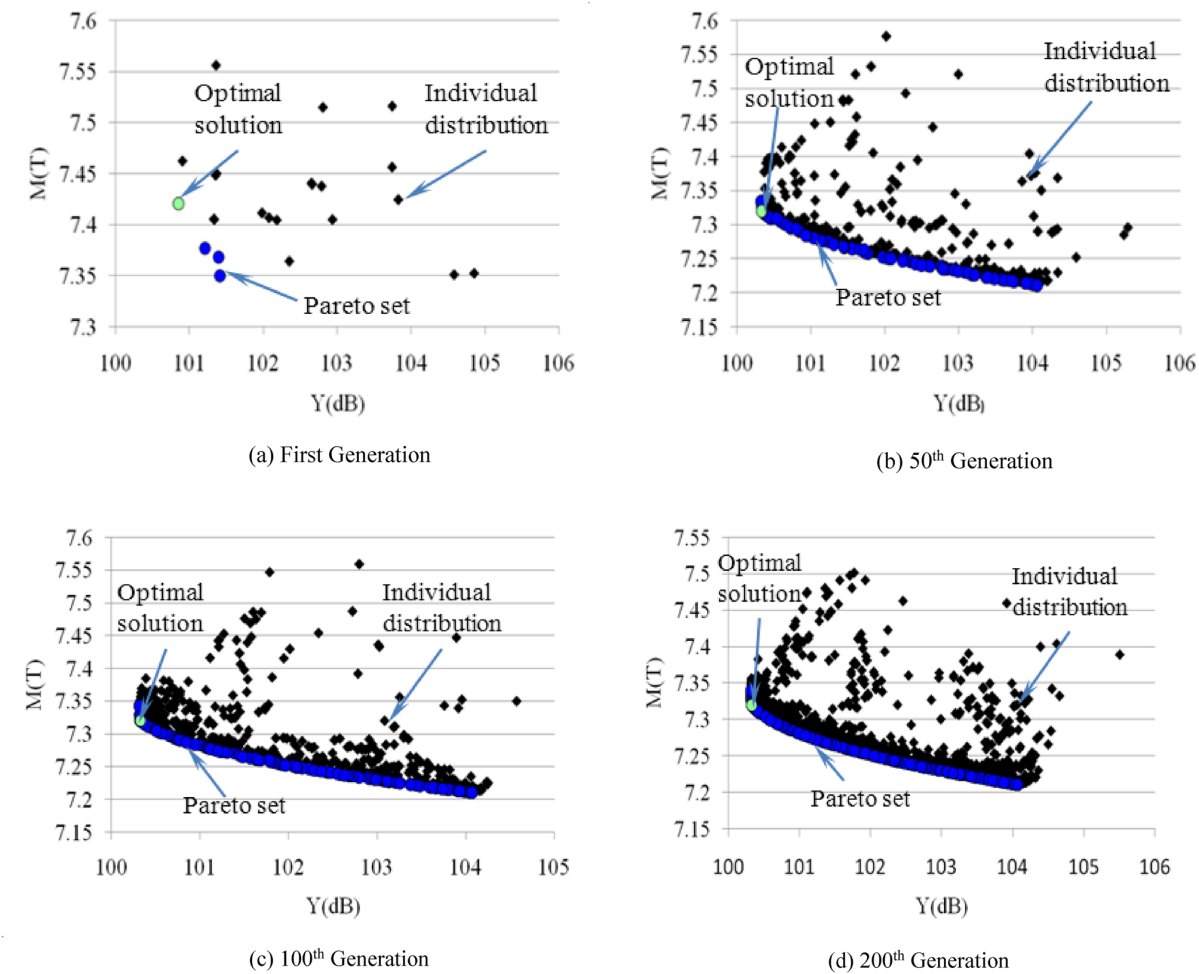
The calculation process of NSGA-II.

The radiated noise, mass of the gear-box housing, and structure of the large-scale planetary gear box can be predicted using the uniform design and response surface model in operation since they are explicit in the quadratic response surface as the objective function of the optimization design. We determined that the variation ranges of the design variables are coincident with the design space of the uniform design, according to the geometric characteristics of the planetary gear box and results of the contribution of the panels, by comprehensively considering the manufacturing technology and structural strength of the gear box, and combining these with the test space of the uniform experimental design. The mathematical model of the optimization design of the large-scale planetary gear box was obtained and is shown below:

25 $$ \left\{ {\begin{array}{ll} {{\text{Find }}\min } & {Y_{dB} (x),M_{mass} (x)} \\ {st} & {M_{mass} (x) \le M_{0} } \\ {x_{ia} \le x_{i} \le x_{ib} } & {i = 1,2,3,4} \\ \end{array} } \right. $$

where $Y_{dB}$ is the maximum sound pressure of the radiated noise, and $M_{mass}$ is the box mass. $M_{0}$ is the original box mass, and $x_{i}$ is the design variable.$x_{ia}$ is the lower limit of the variable, and $x_{ib}$ is the upper limit of the variable. The value range of design variables is shown in Table 4. The NSGA-II algorithm was adopted to solve the multi-objective optimization problem. During the optimization analysis, the size of the initial population was 12, and the genetic algebra was 200 generations. The crossing-over rate was 0.9 and the cross-distribution index was 10, while the distribution index of the variation was 20. Figure 10 shows the evolutionary process of the indexes of the radiated noise sound pressure and box mass. The small dark spot expresses the individual distribution of each generation and the blue dot indicates the solved Pareto solution set. The green dot represents the optimum solution in the Pareto solutions.

**Table 4:** Range of values for design variables.

Variable(mm)	Lower limit	Upper limit
$x_{1}$	0.0	9.0
$x_{2}$	− 4.5	4.5
$x_{3}$	0.0	12.0
$x_{4}$	0.0	6.0

These results show that the population can rapidly converge around the optimum solution set during the evolution process, and the obtained optimum solutions are: × 1 = 9.0 mm, × 2 = -4.5 mm, × 3 = 12.0 mm and × 4 = 6.0 mm. The maximum radiated noise sound pressure value of the field point is 100.33 dB (A), which is obtained by substituting the optimum solutions into the approximate response surface. The optimized box mass is 7,320 kg, and the weight is reduced by 434 kg.

### The strength analysis of the optimized two-stage planetary gear box body

The transient dynamic is re-calculated for the optimized gear box. Thereafter, the structure of gear box and the effective stress of each component at any moment can be obtained and compared with before optimization. It can be seen from Table 5 that the stress of the integral box and each component does not exceed the yield limit. The maximum stress appears to be 149.3 MPa. However, this stress is the local stress located in the strength range of the material. Consequently, the box structure after the response surface optimization can meet the mechanic strength requirement.

**Table 5:** The maximum stress of the box member before and after optimization.

	Front cover in the input end	Front hood	Housing base	Rear cover in the output end
Maximum stress before optimization (MPa)	135.701	107.065	82.063	118.906
Maximum stress after optimization (MPa)	149.333	108.136	114.868	115.897

### The acoustic simulation analysis of the optimized gear box

**Figure 11:**
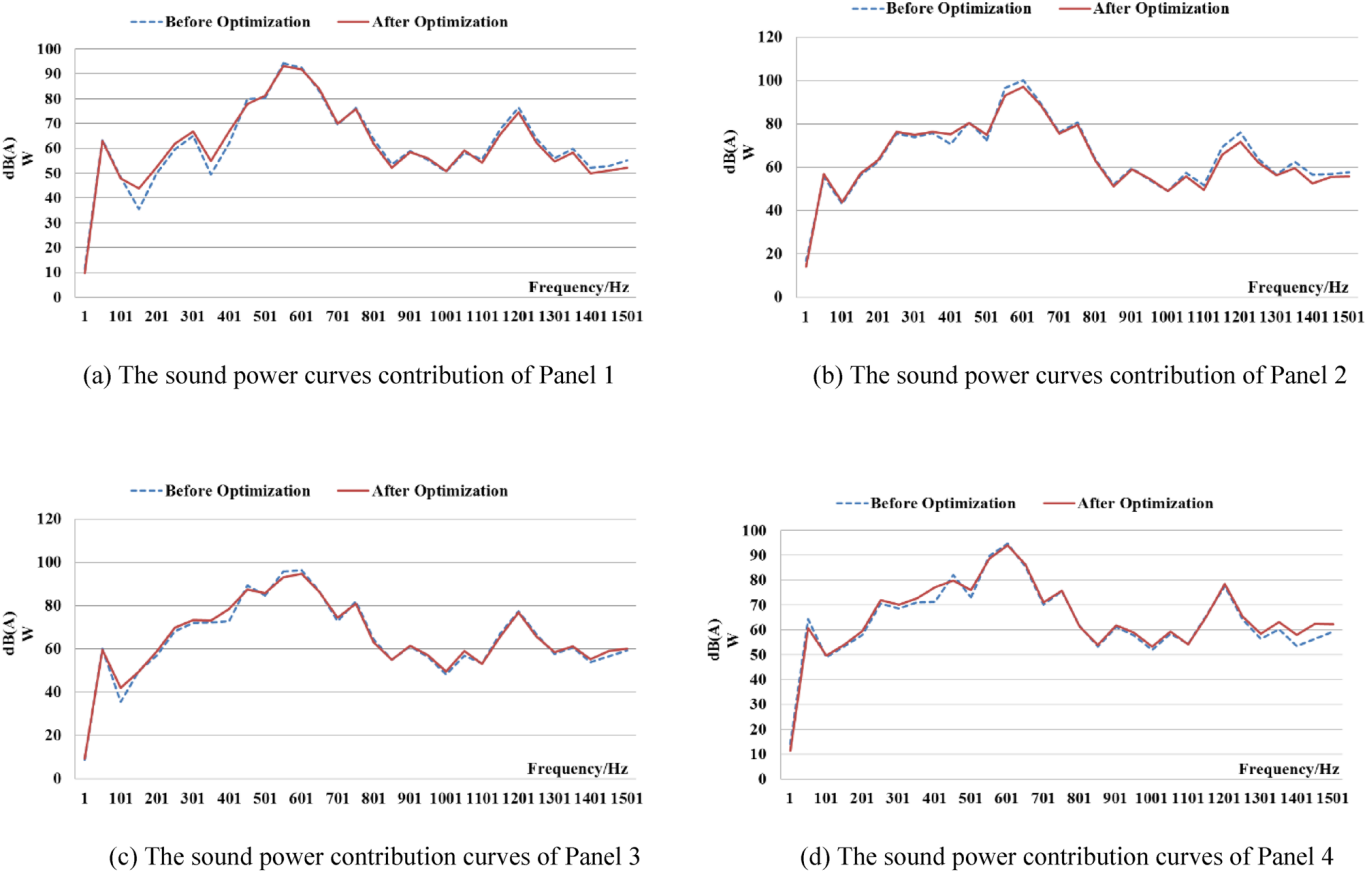
The sound power contribution of the panels before and after optimization.

The design variable values were substituted into the finite element model of the box structure, and the acoustic simulation analysis of the optimized box body was carried out. The sound pressure contribution curves of the main contribution panels before and after the optimization are shown in Fig. 11.

It is evident that the variation trends of the contribution curves of the main contribution panels before and after optimization are coincident in Fig. 11. The sound power contribution of Panel 1 is not greatly changed within 100 Hz. In the frequency range after 450 Hz, the sound power contribution after optimization is slightly lower than before optimization. In the frequency range of 351 Hz–451 Hz, the sound power contribution of the optimized Panel 2 is lower than that before optimization, which indicates that the optimized Panel 2 can suppress the radiated noise of the output end in the reducer. In the 400–700 and other frequency ranges, the sound power contribution of panels 3 and 4 is slightly reduced after optimization.

**Figure 12:**
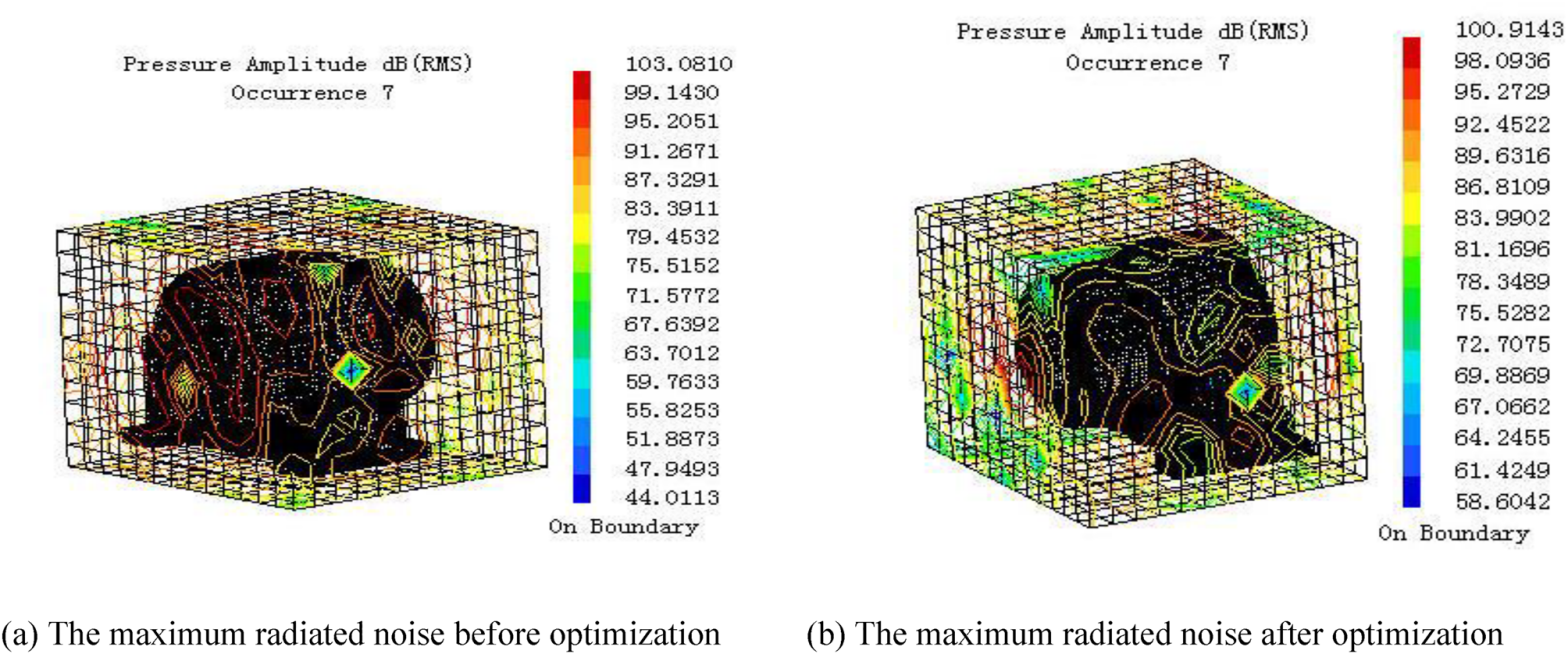
The maximum radiated noise of the box before and after optimization.

As shown in Fig. 12, the radiated noise analysis of the gearbox before and after optimization can obtain that the total sound pressure of the sound power level is reduced from 103.08 dB to 100.91 dB. Thus, it can be inferred that the optimized method can effectively reduce the radiated energy of the gearbox.

### Experimen results for radiation noise of the two-stage planetary gear box body

**Figure 13:**
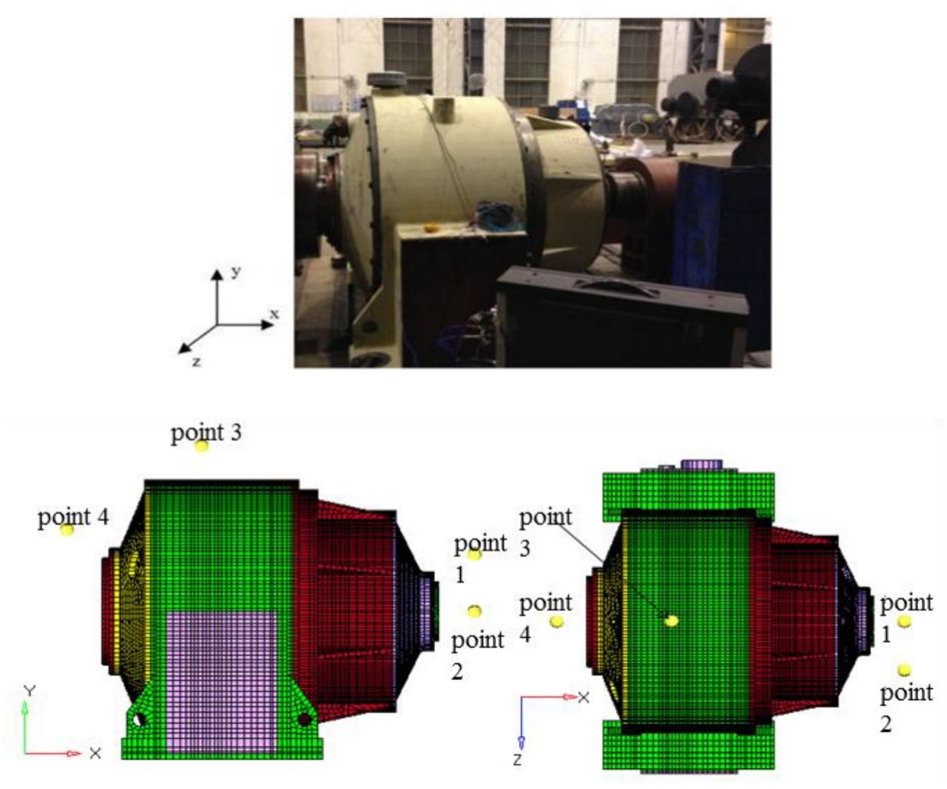
Test rig and radiated noise measuring points.

Figure 13 shows the test bench and testing instrument, including the B&K4190 microphone and the LMS data acquisition instrument, while showing the location of microphone. The experimental conditions are consistent with the theoretical analysis conditions. Table 6 shows the test results of the four test points. The experimental results of the four measuring points are consistent with the simulation results.

**Table 6:** Radiated noise test results.

	Point 1	Point 2	Point 3	Point 4
Test before optimization/dB(A)	102.22	102.33	99.83	100.80
Test after optimization/dB(A)	100.82	100.75	98.47	100.03

## Conclusion

The influence of the large-scale mining equipment on power consumption and the environment is significant. A multi-objective optimization proposal was suggested in which the reductions of a box’s radiated noise and mass are taken as the optimization objectives. The main conclusions are drawn as follows:

1. The prediction analyses of the strength and acoustics under the rated working conditions were undertaken using the well-established transient dynamic analysis model for a system-level planetary gear box. The uniform experimental method was adopted to perform the experiment design, which resolved the problem of handling a large number of calculations. The multi-objective optimized mathematical model of the planetary gear box was established as a function of the design variables and constraint conditions.
2. The optimization solution of the multi-objective response was carried out using the NSGA-II optimization algorithm, and the optimized design scheme of the box body was proposed. The optimization results were shown the mass of the house of gear box was reduced by 434 kg, and its maximum sound pressure of the radiated noise by 2.33 dB(A).

## Data Availability

The datasets generated during and/or analyzed during the current study are available from the corresponding author on reasonable request.

## References

[1] Seewoogolam V, Prasad B, Manral AR, Alarifi IM, Asmatulu R (2022). Modal analysis and improvement of lightweight wings for micro air vehicle (Mav) applications. J. Mech. Sci. Technol..

[2] Pfaffa BM, Hoschke K (2020). Resource analysis model and validation for selective laser melting, constituting the potential of lightweight design for material efficiency. Sustain. Prod. Consump..

[3] Schleinkofer U, Laufer F, Zimmermann M, Roth D, Bauernhansl T (2018). Resource-efficient manufacturing systems through lightweight construction by using a combined development approach. Procedia CIRP.

[4] Wang M, Dong J, Wang W (2013). Optimal design of medium channels for water-assisted rapid thermal cycle mold using multi-objective evolutionary algorithm and multi-attribute decision-making method. Int. J. Adv. Manuf. Technol..

[5] Hadi M, Mahmood G, Azizi R (2014). Constrained grinding optimization for time, cost, and surface roughness using NSGA-II. Int. J. Adv. Manuf. Technol..

[6] Daroczy L, Gabor J, Thevenin D (2014). Systematic analysis of the heat exchanger arrangement problem using multi-objective genetic optimization. Energy.

[7] Kia K, Fitch SM, Newsom SA, Kim JH (2020). Effect of whole-body vibration exposures on physiological stresses: mining heavy equipment applications. Appl. Ergon..

[8] Deng X, Dong J, Wang S (2022). Reducer lubrication optimization with an optimization spiking neural P system. Inf. Sci..

[9] Schönknecht A, Babik A, Rill V (2016). Electric powertrain system design of BEV and HEV applying a multi objective optimization methodology. Transp. Res. Procedia.

[10] Cao Z, Xia J, Zhang M (2015). Optimization of gear blank preforms based on a new R-GPLVM model utilizing GA-ELM. Knowl.-Based Syst..

[11] Wang J, Luo S, Su D (2016). Multi-objective optimal design of cycloid speed reducer based on genetic algorithm. Mech. Mach. Theory.

[12] Zhao X, Yan X, Chen Z (2021). Lightweight design and dynamics analysis of ZYL-15000D directional drill reducer. Adv. Maer. Sci. Eng..

[13] Slavov S, Konsulova-Bakalova M (2019). Optimizing weight of housing elements of two-stage reducer by using the topology management optimization capabilities integrated in SOLIDWORKS: A case study. Machines.

[14] Tambolia K, Patel S, George PM, Sanghvi R (2014). Optimal design of a heavy duty helical gear pair using particle swarm optimization technique. Procedia Technol..

[15] Choi DH, Moon IS, Choi BK (2004). Effects of sub-antimicrobial dose doxycycline therapy on crevicular fluid MMP-8, and gingival tissue MMP-9, TIMP-1 and IL-6 levels in chronic periodontitis. J. Periodont. Res..

[16] Ide T, Otomori M, Leiva J, Brian C (2014). Watson. Structural optimization methods and techniques to design light and efficient automatic transmission of vehicles with low radiated noise. Struct. Multidiscip. Optim..

[17] Samad A, KiM KY (2008). Multi-objective optimization of an axial compressor blade. J. Mech. Sci. Technol..

[18] Kimlt JH, Choi JH, Husain A (2010). Performance enhancement of axial fan blade through multi-objective optimization techniques. J. Mech. Sci. Technol..

[19] Wang W, Mo R, Zhang Y (2011). Multi-objective aerodynamic optimization design method of compressor rotor based on Isight. Procedia Eng..

[20] Miler D, Žeželj D, Lončar A, Vučković K (2018). Multi-objective spur gear pair optimization focused on volume and efficiency. Mech. Mach. Theory.

[21] Narayanan RC, Ganesh N, Jangir P (2023). A novel many-objective sine-cosine algorithm (MaOSCA) for engineering applications. Mathematics.

[22] Booth DN, Kohar CP, Inal K (2021). Multi-objective optimization of a multi-cellular aluminum extruded crush rail subjected to dynamic axial and oblique impact loading conditions. Thin-Walled Struct..

[23] Zendehboudi A, Li X (2018). Desiccant-wheel optimization via response surface methodology and multi-objective genetic algorithm. Energy Convers. Manag..

[24] Yang W, Tang X (2021). Research on the vibro-acoustic propagation characteristics of a large mining two-stage planetary gear reducer. Int. J. Nonlinear Sci. Numer. Simul..

